# Observation of collective excitation of surface plasmon resonances in large Josephson junction arrays

**DOI:** 10.3762/bjnano.13.132

**Published:** 2022-12-28

**Authors:** Roger Cattaneo, Mikhail A Galin, Vladimir M Krasnov

**Affiliations:** 1 Stockholm University, Physics Department, SE-10691 Stockholm, Sweden; 2 Institute for Physics of Microstructures RAS, 603950 Nizhny Novgorod, Russia

**Keywords:** cavity modes, Josephson junctions, synchronization mechanism, THz radiation

## Abstract

Josephson junctions can be used as sources of microwave radiation. However, synchronization of many junctions is required for achieving a coherent amplification of the emitted power. In this work we present an experimental study of large arrays containing up to one thousand Nb/Nb*_x_*Si_1−_*_x_*/Nb junctions. The arrays exhibit profound cavity mode resonances, corresponding to the formation of standing waves at the electrode/substrate interface. We observe that resonant steps in the current–voltage characteristics appear above some threshold number of junctions, *N*_th_ ≈ 100, and then progressively enhance in amplitude with further increment of the number of junctions in the resistive oscillating state. We use an external detector to measure the emission of electromagnetic waves. The emission power correlates with the step amplitude. Our results indicate that the emission is facilitated by the cavity modes in the electrodes. The modes are collectively excited by active junctions. In turn, the standing wave imprints its order on the array, facilitating mutual phase-locking of junctions. This provides an indirect coupling mechanism, allowing for the synchronization of junctions, which do not directly interact with each other. Our results demonstrate that electrodes can effectively work as a common external resonator, facilitating long-range phase-locking of large junction arrays with sizes larger than the emitted wavelength.

## Introduction

Terahertz sources of electromagnetic waves (EMWs) in the range of 0.1–10 THz are characterized by a low power efficiency [1–6]. Josephson junctions (JJs) can generate tunable terahertz radiation in a broad frequency range, from sub-terahertz in low-*T*_c_ superconductors [7–9], to tens of terahertz in high-*T*_c_ superconductors [10–18]. The performance of Josephson oscillators is limited by impedance mismatch [18–19] and self-heating [13,17,20–21]. Proper device engineering can obviate these obstacles and improve the performance [18]. A single JJ is able to emit EMWs, but with a low power [22]. Therefore, synchronization of many JJs is required for coherent superradiant amplification of the emitted power [7–10,19,23–25].

Synchronization of many oscillators is a difficult task. It requires their mutual coupling, which can be either direct or indirect. The direct coupling is caused by interjunction interaction via shared electromagnetic fields and currents [26]. For conventional overlap JJs the scale of such interaction is short (nanoscale) because it is limited by the corresponding screening lengths in superconducting electrodes [27]. However, for planar JJs the direct interaction can be of long range due to the presence of long-range stray fields [28]. Indirect coupling is caused by interaction of JJs with a common external resonator [7–8,29–35]. The resonator imprints the phase order onto the junction array and, thus, can synchronize JJs without direct interjunction coupling. The scale of such indirect coupling is not limited by screening lengths and can be truly of long range. For example, in [9,34], the successful indirect synchronization of up to 9000 JJs in a large array (≈1 cm) was demonstrated. The indirect coupling via a common resonator is the most promising way for the synchronization of very large arrays with sizes significantly larger than the emitted wave length.

In this work, we study experimentally large JJ arrays containing up to 1000 Nb/Nb*_x_*Si_1−_*_x_*/Nb junctions. The arrays exhibit strong cavity mode resonances resulting in the appearance of a profound step structure in the current–voltage (*I*–*V*) characteristics. The resonances are caused by the formation of surface plasmon-type standing waves at the electrode–substrate interface [34]. Thus, the electrodes themselves act as a common external resonator, facilitating the effective indirect coupling between JJs and the long-range phase-locking of the arrays. Our main result is the observation of a gradual development of collective resonances upon sequential switching of JJs into the oscillating resistive state. We show that a threshold number of JJs, *N*_th_ ≈ 100, is required for excitation of the collective cavity modes. Above the threshold, the amplitude of resonant steps grows in a quasi-linear manner with the number of active JJs. We employ an external microwave detector for measuring EMW emission from the arrays. It is observed that the emitted power is correlated with the amplitude of the resonant step in the *I*–*V*s, implying that the emission is facilitated by the cavity modes [9,32–34]. We conclude that the long electrodes in the studied arrays are acting both as external resonators and microwave antennas. The large length (approx. 1 cm) of the electrodes facilitates good impedance matching with free space and improves the radiation power efficiency. The cavity modes in the electrodes are excited collectively by the JJs, which are, in turn, mutually phase-locked by the modes. This provides a positive feedback mechanism allowing for the synchronization of large arrays without direct interjunction interaction.

## Samples

**Figure 1:**
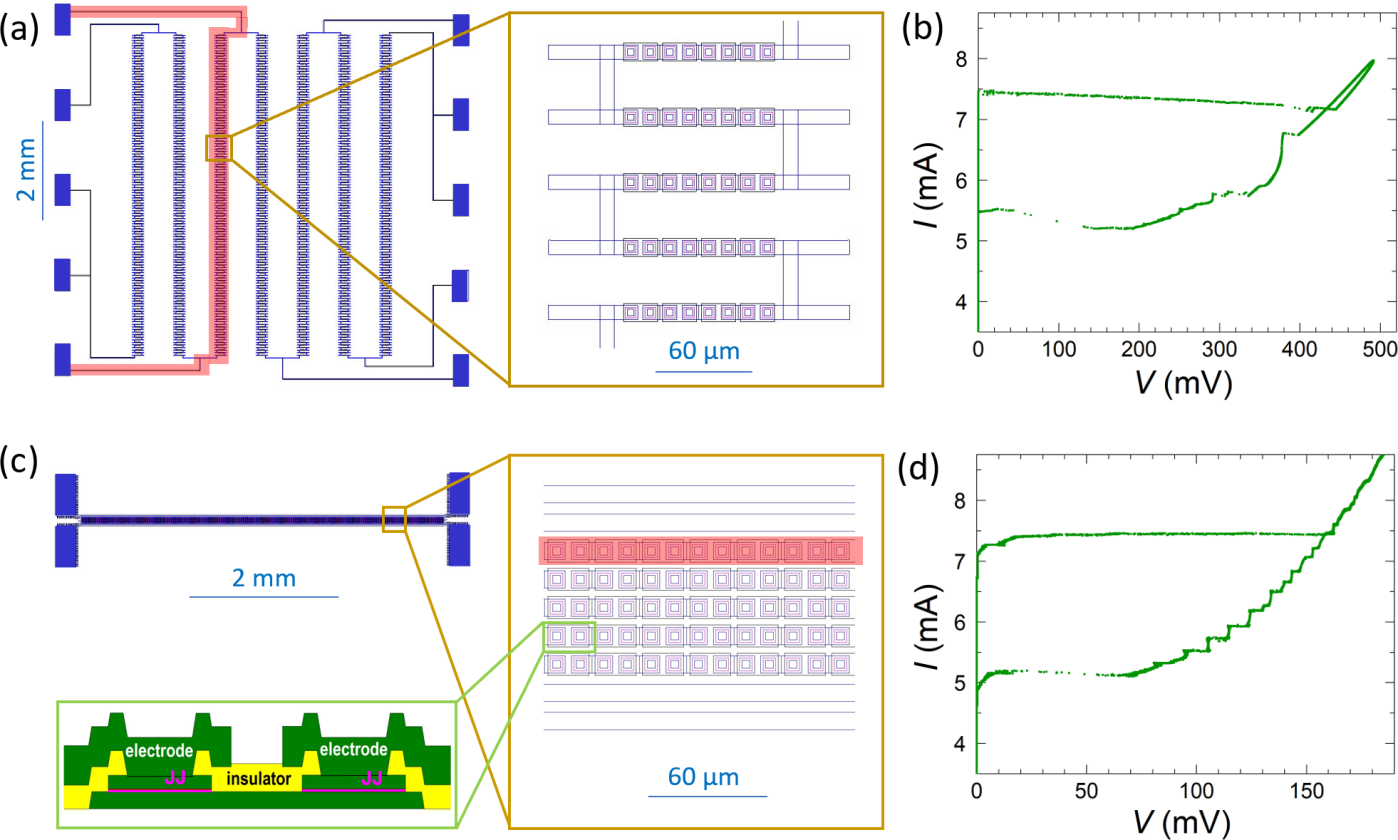
(a, c) Layouts of the studied meander and linear arrays. The meander array in (a) contains seven meandering sections with *N* = 1000 JJs in each. The linear array in (c) contains five straight lines with *N* = 332 JJs in each. The right panels represent corresponding closeups (top view). The bottom-left part in (c) shows a sketch of the junction cross section with Nb in green, SiO_2_ in yellow, and the junction interlayer in magenta (from [36]). Red bands in (a) and (c) mark one of the sections in each array, which was used for detailed studies. Panels (b) and (d) show the *I*–*V* characteristics of the marked sections in the meander (b) and linear (d) arrays. Measurements are done in zero magnetic field at *T* ≃ 2.6 K. Appearance of profound steps in the *I*–*V*s due to excitation of cavity resonances in electrodes is clearly seen for both arrays.

We study arrays of Nb/Nb*_x_*Si_1−_*_x_*/Nb JJs fabricated on oxidized Si substrates and connected in series by Nb electrodes. The Nb*_x_*Si_1−_*_x_* interlayer with the composition *x* ≃ 0.14 was deposited by co-sputtering from Nb and Si sources. Details about the fabrication procedure can be found in [36]. Figure 1a and Figure 1c show layouts of the two studied arrays, which we refer to as (a) “meander” and (c) “linear”, respectively. The arrays are similar to those studied in [9,34,37], but have smaller JJ areas. Additional information about transport properties of such arrays can be found in [9,34,36–37].

The meander array (Figure 1a) has seven identical (vertical) sections with *N* = 1000 JJs in each and with an overall size in the vertical direction of 6.25 mm. Each section consists of 125 horizontal segments with a length of 230 μm, a width of 10 μm, and a separation of 40 μm. Each segment contains eight overlap-type JJs with the area 6 × 6 μm^2^. This can be seen from the close-up shown in the left panel of Figure 1a. The distance between nearby junction centers is 12 μm. A cross section of the junctions is sketched in the bottom-left panel of Figure 1c.

The linear array (Figure 1c) contains five straight sections (lines) with *N* = 332 JJs in each. The spacing between the lines is 6 μm. Sizes and separation of the JJs is the same as for the meander array.

The two outermost lines in the linear array and all seven sections of the meander array can be measured independently. We tested all of them, and they show similar characteristics. Below we will show data for one of the sections of each array, marked by the red bands in Figure 1a and Figure 1c. The meander array does not contain any specific resonator. In this case, collective cavity modes originate solely from interconnecting Nb electrodes, acting as a travelling wave antenna for surface plasmons at the electrode–substrate interface [9,34]. The linear array contains also two extra Nb lines (without JJs) on each side of the array forming a slot waveguide, which may act as an additional external resonator. However, comparison with similar arrays without such lines [9,34] does not reveal any significant influence of these lines, implying that even for the linear array the electrodes are playing the dominant role in array dynamics.

## Results

Measurements were performed in a closed-cycle ^4^He cryostat (sample in gas) with rotatable sample holder. The magnetic field is supplied by a superconducting solenoid. Figure 1b and Figure 1d show the *I*–*V* curves (up and down bias swipes) for meander and linear arrays, respectively, at zero magnetic field and *T* ≃ 2.6 K. For both arrays, all JJs switch simultaneously from the superconducting to the resistive state, at similar critical currents, *I*_c_ ≃ 7.5 mA. This indicates good reproducibility of the fabrication procedure with almost identical JJs. The characteristic voltage per JJ is quite large, *I*_c_*R*_n_ ≃ 0.5 mV. As seen from Figure 1b and Figure 1d, well-defined vertical steps appear in the reverse branch of the *I*–*V* characteristics for both arrays. As shown in [9,34], they are caused by propagation of surface plasmon-type EMWs along the Nb electrode–Si substrate interface. These steps appear when the Josephson frequency coincides with one of the cavity mode frequencies, corresponding to formation of standing waves along the whole length (approx. 1 cm) of the electrodes [34]. Cavity modes depend on the array geometry. Therefore, the step structure is different for the two arrays. The meander array, Figure 1b, exhibits a single large step at high voltage and many low-amplitude steps with small separation in voltage. The linear array, Figure 1d, exhibits several evenly spaced steps. The *I*–*V* characteristics are hysteretic, with the retrapping current being significantly smaller than the switching current. The hysteresis leads to a metastability, which allows for the observation of different voltage states at the same current. This will be exploited for accessing a larger variety of states with different number of active junctions in the oscillating resistive state.

**Figure 2:**
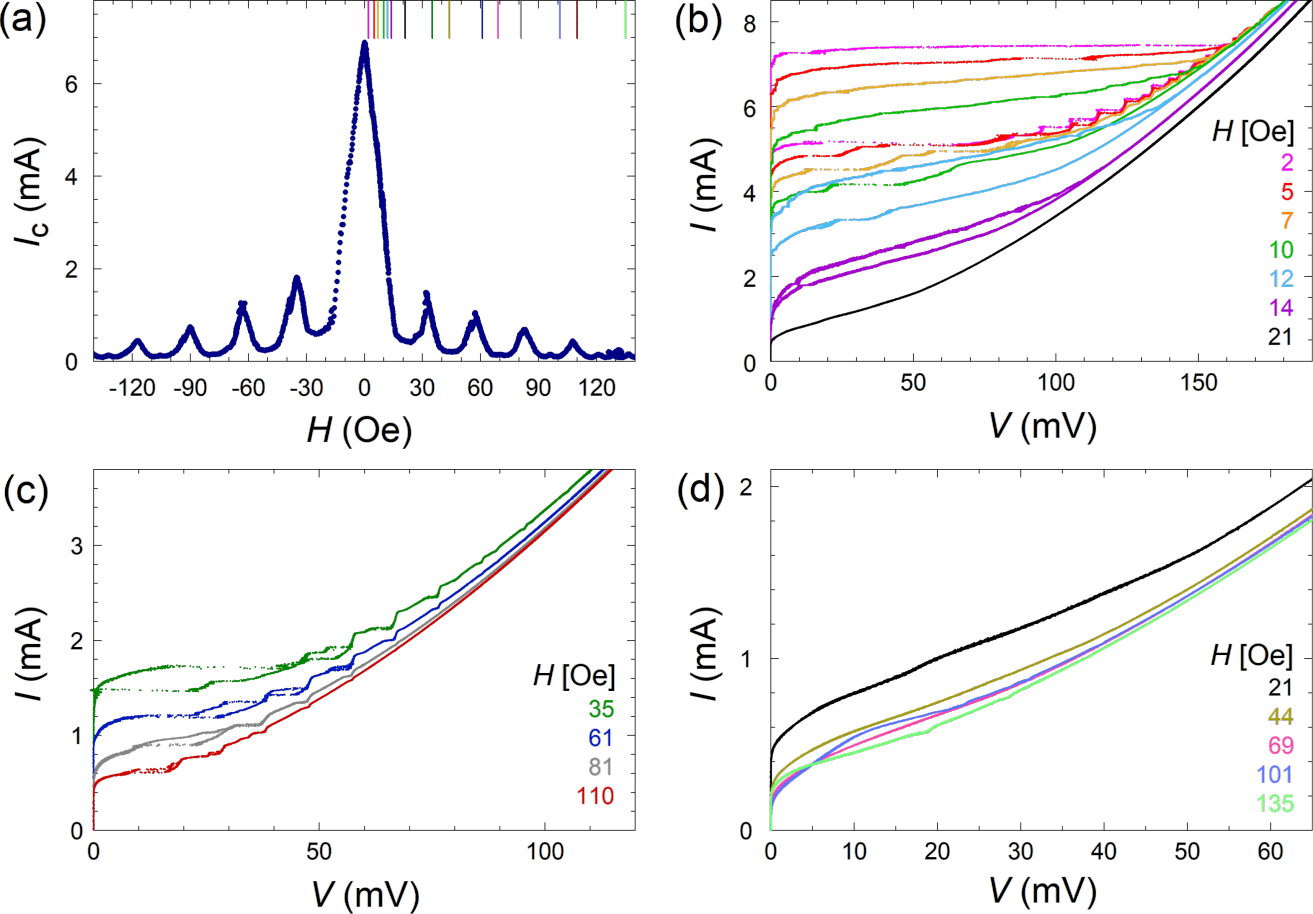
(a) Fraunhofer-type modulation of the critical current as a function of in-plane magnetic field for the linear array. Panels (b–d) show the *I*–*V* characteristics at different magnetic fields indicated by the same-color vertical lines in (a). Panel (b) represents the *I*–*V*s at the central lobe. Panels (c) and (d) show the *I*–*V*s close to (c) maxima and (d) minima of *I*_c_(*H*) at different lobes. A correlation between *I*_c_ and the step amplitudes can be seen.

Figure 2a shows the modulation, *I*_c_(*H*), of the critical current versus the in-plane magnetic field for the linear array. The period of modulation agrees well with the expected value for a single JJ. However, the shape of *I*_c_(*H*) with sharp peaks deviates from the standard Fraunhofer pattern, characteristic for a single JJ. The reason is that the measured *I*_c_ represents the smallest *I*_c_ for all JJs in the array. As discussed below, a magnetic field causes a spread in the modulations *I*_c_ for different JJs. Therefore, the measured *I*_c_(*H*) is lower than the Fraunhofer modulation for an individual JJ. Presumably, the spread of the modulations *I*_c_ is caused by the uneven distribution of fluxon numbers in JJs when the flux per junction is not equal to integer and half-integer numbers of flux quanta.

Figure 2b–d shows the *I*–*V*s measured at different magnetic fields indicated by vertical lines with the corresponding color in Figure 2a. The step amplitude, Δ*I*, modulates in a correlated manner with *I*_c_(*H*). Δ*I* is large at maxima of *I*_c_(*H*), see Figure 2c, and vanishes when *I*_c_(*H*) → 0, see Figure 2d. The correlation between Δ*I* and *I*_c_ reflects the cavity mode–junction interaction. The mechanism for appearance of resonant steps is similar to that for the formation of Shapiro steps upon external microwave (MW) irradiation. The steps appear via rectification of the cavity-mode-induced MW current in the electrodes and the amplitude of the rectified current is proportional to *I*_c_ of the JJs [38].

### Dynamic states with different number of active junctions

We want to emphasize that the discussed cavity resonances represent a collective behavior of the arrays. A single JJ does not exhibit such steps [36]. To analyze the collective behavior, it is necessary to switch junctions one-by-one into the oscillating resistive state. Since the JJs are almost identical, at *H* = 0, they all simultaneously switch together, as seen in Figure 1b and Figure 1d. However, application of a small magnetic field introduces a spread in critical currents, as can be seen from Figure 2b. In combination with the hysteresis in the *I*–*V* characteristics, this makes it possible to reach a large variety of dynamic states with different numbers of active JJs.

**Figure 3:**
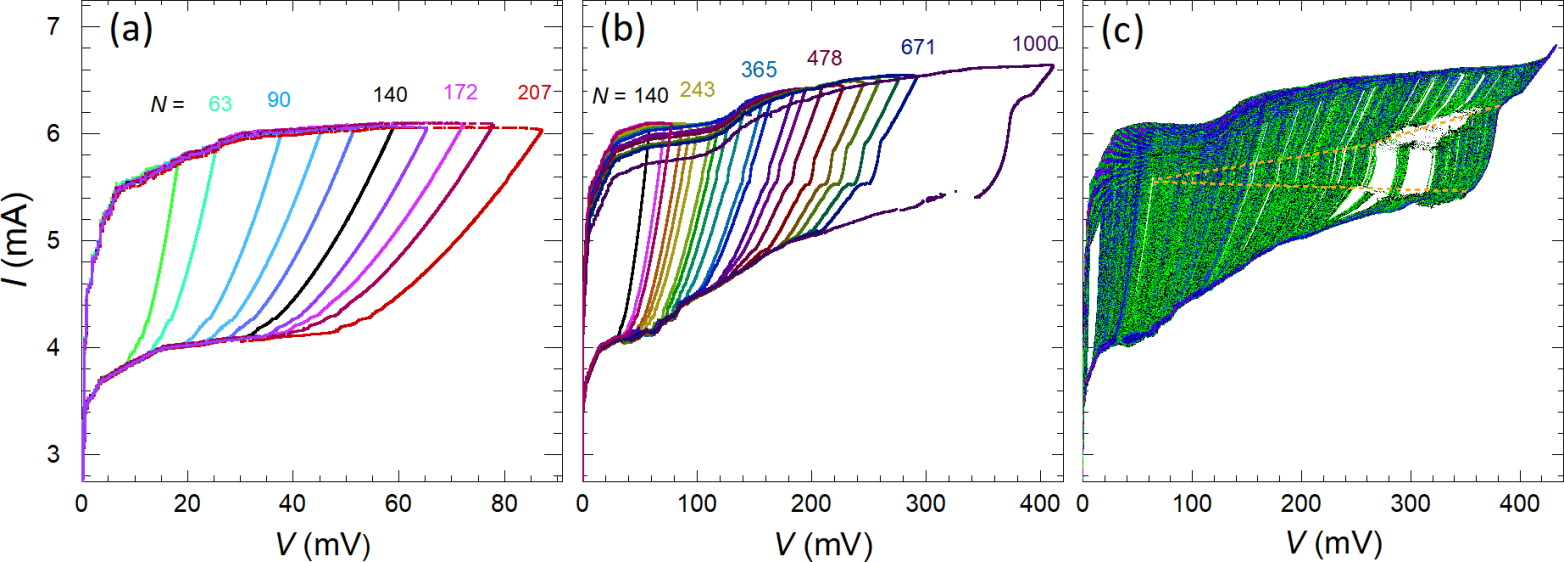
Ensembles of the *I*–*V* characteristics with different numbers of active JJs for the meander array at *T* = 2.4 K and for an in-plane field of *H* = 7 Oe. (a, b) The *I*–*V*s obtained in a single swipe (a) for a small number of JJs, *N* ≤ 207 and (b) at larger *N* = 140–1000. The number of active JJs, *N*, is indicated for some *I*–*V*s using the same color. A gradual development of the main resonant step at *I* ≃ 5.5 mA with increasing *N* can be seen. (c) An integrated oscillogram accumulated during many current swipes with varying amplitudes. It demonstrates a large variety of achievable states with different numbers of active JJs. Dashed orange lines indicate a quasi-linear increase of the step amplitude with increasing *N* for *N > N*_th_ ≃ 150.

Figure 3a and Figure 3b show thus obtained *I*–*V*s of the meander array with different number of active JJs. Here, each *I*–*V* curve is measured in a single swipe while sequentially increasing the current amplitude. Figure 3a and Figure 3b represent the *I*–*V*s for small and large ranges of *N*, respectively. The voltage at a given current scales linearly with the number of JJs, which allows for an unambiguous determination of *N*. In Figure 3a, *N* = 46, 63, 90, 107, 122, 140, 155, 172, 184, and 207 (from left to right). In Figure 3b *N* = 140, 172, 184, 207, 224, 243, 271, 284, 304, 328, 365, 380, 422, 444, 478, 521, 560, 591, 632, 671, and 1000 (all JJs). The number of active junctions for some curves is indicated by the corresponding color. As can be seen from Figure 3a, the *I*–*V* characteristics for a small number of JJs in the resistive state do not exhibit resonant steps. In Figure 3b, the steps are gradually developing above some threshold number of JJs, *N*_th_. Figure 3c represents an integrating oscillogram measured during repetitively sweeping the bias up and down with slowly changing amplitude. It allows for an almost complete mapping of the *I*–*V* evolution with changing the number of active JJs. Dashed orange lines indicate edges of the main resonance step. They reveal a quasi-linear increase of the step amplitude with increasing *N*, for *N > N*_th_ ≃ 150.

**Figure 4:**
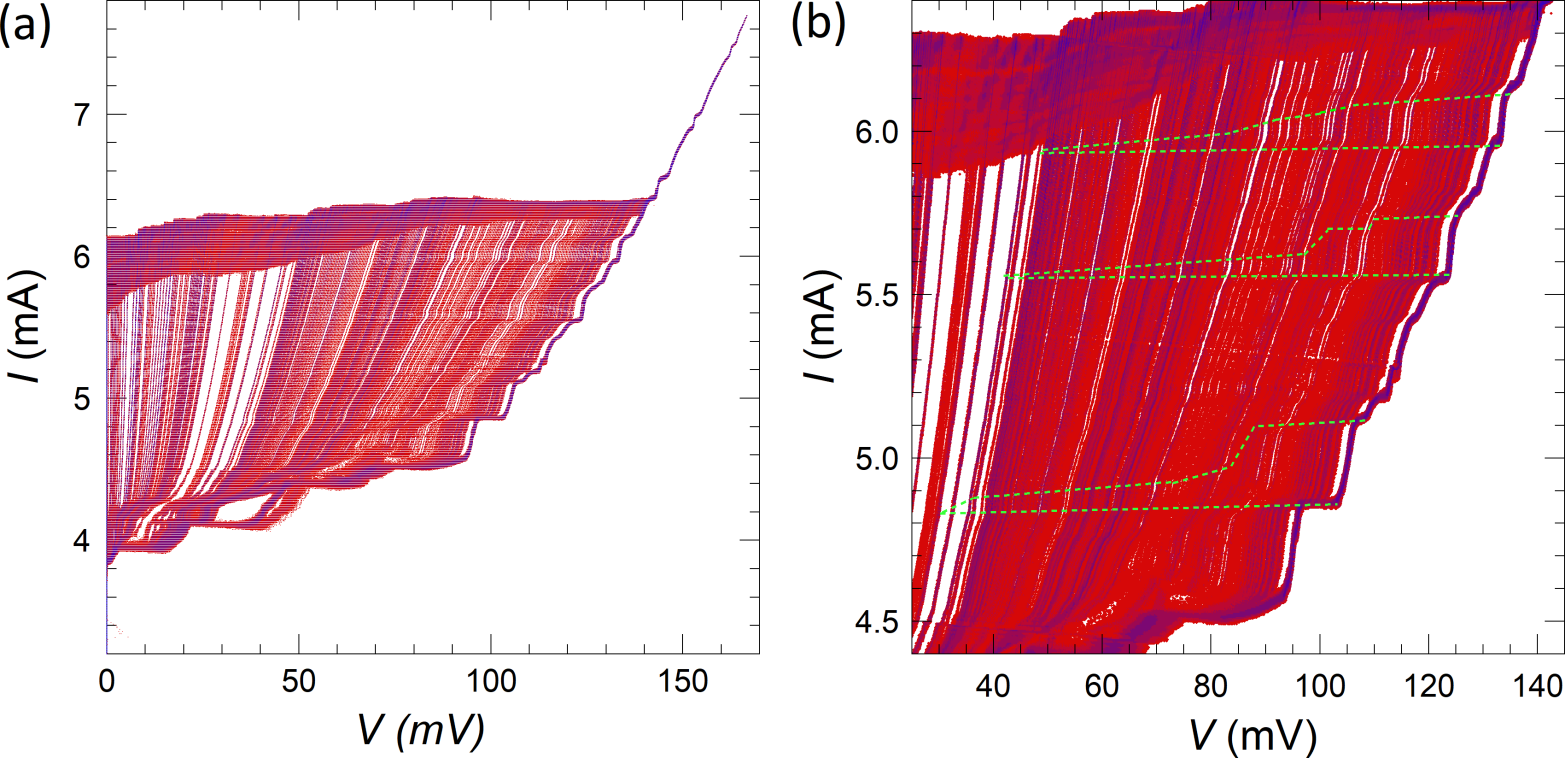
(a) An integrated oscillogram for the linear array at *T* = 2.4 K and for an in-plane field *H* = 7 Oe. Panel (b) shows a closeup view, which illustrates appearance and gradual evolution of several steps with increasing *N*. Edges of three distinct resonant steps are indicated by dashed green lines.

Figure 4 shows similar data for the linear array. Figure 4a shows the integrating oscillogram of the *I*–*V* obtained at *H* ≃ 7 Oe. Figure 4b shows a close-up of the step structure. Dashed green lines in Figure 4b mark edges of three distinct cavity modes. The general behavior is the same as for the meander array. We observe the appearance and the gradual increase of the step amplitudes at *N > N*_th_. The threshold numbers for the three highlighted steps are *N*_th_ ≃ 88, 113, and 120 for the low-, middle-, and high-voltage resonances, respectively. Due to the presence of many closely spaced resonant steps in the *I*–*V* of the linear array, it is difficult to identify and analyze specific modes. Therefore, in what follows, we will perform a quantitative analysis only on the meander array.

**Figure 5:**
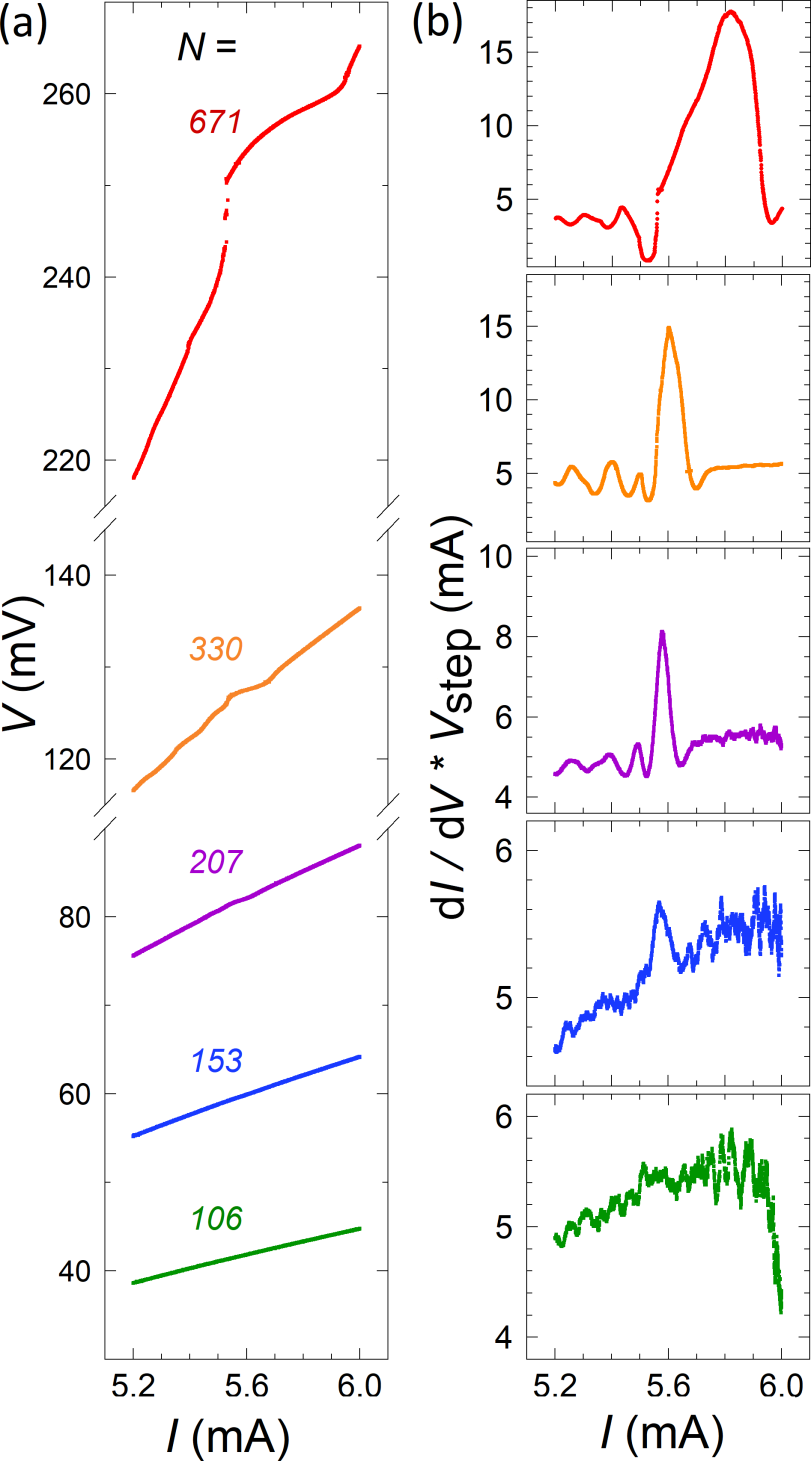
(a) Parts of the *V*–*I* curves near the main resonance for the meander array with different number of active JJs, *N*. (b) Normalized differential conductance for the *I*–*V* characteristics from (a). The peak represents the resonant step. It reduces with decreasing *N* and is not visible for *N* = 106 (lower panel) below the threshold number of JJs.

In Figure 5a, we show portions of the *I*–*V* curves for the meander array with different number of active JJs in the bias range corresponding to the main resonance. The resonant step is large for *N* = 671 (red), distinct for *N* = 330 (orange), barely visible for *N* = 207 (violet), and not seen for smaller *N*. To quantify the step amplitude, Δ*I*, at small *N* we plot the differential conductance, d*I*/d*V*. Figure 5b shows the d*I*/d*V*(*I*) curves for the *I*–*V*s from Figure 5a, normalized by the resonant voltage *V*_step_. This quantity does not depend on the number of active JJs. A current step in the *I*–*V* is represented by a peak in *V*_step_ d*I*/d*V*. The height of the peak reflects the sharpness of the step and the width is equal to the step amplitude, Δ*I*. For the well-developed step, *N* = 671, both the height and the width of the peak are large. From Figure 5b, it can be seen that with decreasing *N* both the height and the width of the peak decrease. For *N* = 153 (blue) a small peak is still visible, but for *N* = 106 (olive) we can not distinguish any signature of the resonant step. This observation clearly shows that the observed steps are not inherent to individual Nb/Nb*_x_*Si_1−_*_x_*/Nb JJs but are the consequence of collective surface-plasmon resonances. The finite threshold number is the consequence of the collective excitation of the cavity mode [32–33].

### Radiation detection

**Figure 6:**
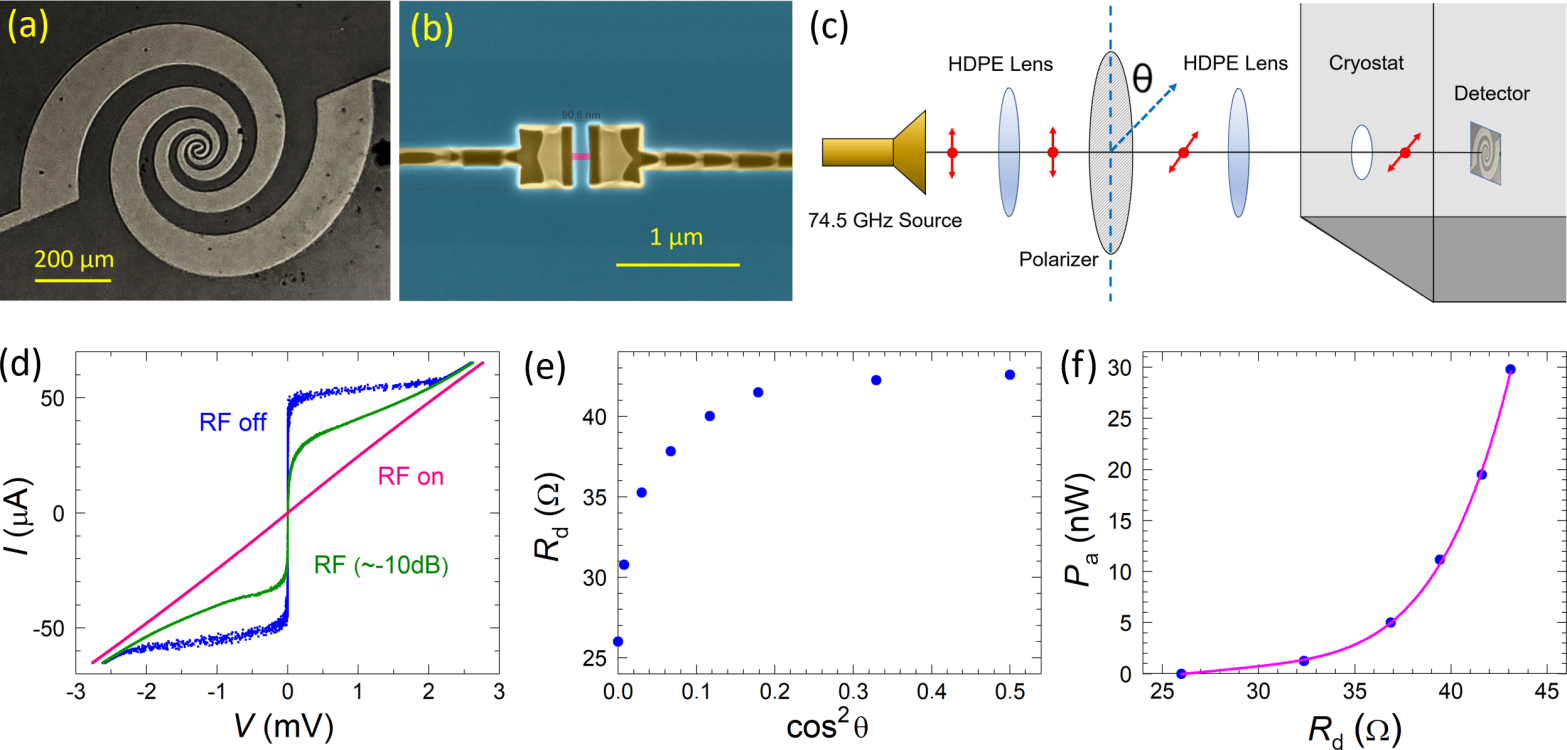
(a) Optical image of a superconducting detector with a log-periodic microwave antenna. (b) SEM image of a nanoscale sensor junction (false color). (c) Schematics of the calibration setup using a microwave source with *f* = 74.5 GHz. (d) The *I*–*V* characteristics of the sensor junction at *T* = 2.4 K without (blue) and with attenuated (olive) and full (pink) microwave power. (e) The ac resistance of the detector, *R*_d_, as a function of cos^2^θ of the polarizer angle, proportional to the transmitted power. (f) Obtained calibration curve of the detector, that is, absorbed microwave power, *P*_a_, as a function of *R*_d_.

For detection of EMW emission we use a superconducting microwave detector. Figure 6a shows an optical image of the detector. It consists of a log-periodic microwave antenna [39] with a broad frequency range of ca. 15–700 GHz. In the center, there is a nanoscale JJ sensor, shown in Figure 6b. The detector is made of a Nb film (70 nm thick), using a fabrication technique similar to that described in [28]. The antenna is patterned using photolithography and reactive ion etching. The JJ sensor with variable thickness and a width of ≈100 nm is made by Ga^+^ focused ion beam etching. The JJ is made small in order to increase its resistance *R*_n_ to approx. 50 Ω, which is needed for a good impedance matching with the antenna.

In order to calibrate the detector, we use a MW source with *f* = 74.5 GHz. Figure 6c represents a sketch of the calibration setup. The detector is placed in an optical cryostat and the gigahertz signal is guided through an optical window using two high-density polyethylene lenses. The gigahertz source is linearly polarized. To tune the incoming microwave power, *P*_MW_, we use a wire-grid polarizer with adjustable angle θ. The blue curve in Figure 6d represents the *I*–*V* of the detector without MW power. It is seen that the detector JJ has a very large *I*_c_*R*_n_ ≃ 2 mV, enabling large readout signal and broad operation frequency up to *f* ≈ 1 THz. Olive and pink curves in Figure 6d show the *I*–*V*s with attenuated and full MW power, respectively. The detector operates as a Josephson switching current detector [15]. The incoming MW signal suppresses the switching current, *I*_s_, of the detector JJ. At high MW power, it is fully suppressed and the *I*–*V* is Ohmic with a normal resistance *R*_n_ = 42.8 Ω, as seen from the pink *I*–*V* from Figure 6d.

To quantify the suppression of *I*_s_, we measure the ac resistance at a fixed current amplitude, *I*_ac_, corresponding to the bias range in Figure 6d (without dc bias offset). As shown in [40], *R*_ac_/*R*_n_ ≃ 1 − (*I*_s_/*I*_ac_)^2^. Figure 6e shows the thus obtained detector resistance, *R*_d_, versus cos^2^θ of the polarizer, which is proportional to the incoming MW power. With increasing *P*_MW_, *R*_d_ decreases due to suppression of *I*_s_. At high MW power, when *I*_s_ → 0, *R*_d_ → *R*_n_ = 42.8 Ω. As discussed in [15], the complete suppression of *I*_s_ corresponds to the absorbed MW power, *P*_a_ ≃ 
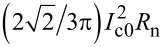
 ≃ 30 nW. Thus, we can perform an absolute calibration of the absorbed power as a function of the detector resistance, as shown in Figure 6f. The absorbed power depends on the absorption efficiency, γ, the antenna area, *A*_a_, and the power density, *P*_MW_/*A*_MW_, where *A*_MW_ is the microwave spot area, *P*_a_ = γ*P*_MW_(*A*_a_/*A*_MW_). Taking an optimal γ = 0.5 [19,41] and an antenna radius of *r*_a_ ≃ 0.5 mm while also assuming a MW spot radius of *r*_MW_ ≈ 1 cm, we can estimate the total incoming power, *P*_MW_ = γ^−1^*P*_a_(*r*_MW_/*r*_a_)^2^ ≃ 24 μW. It is consistent with the total power of the source of approx. 100 μW, taking into account losses on the way to the detector via diffraction on optical windows and a set of low-temperature MW filters (Zitex). Such an estimation indicates that the absorption efficiency γ of our detector is indeed not far from the optimal. However, the overall detection efficiency, *P*_a_/*P*_MW_ ≈ 10^−3^, is small because of geometrical constraints, (*r*_a_/*r*_MW_)^2^ ≪ 1.

For the analysis of EMW emission, the detector is placed face-to-face at about 1.5 cm distance from the array. The sample/detector arrangement is similar to that described in [9]. The detector position is fixed, but the sample is placed on a rotatable sample holder, facilitating adjustment of the angle α between the normals of the detector and the array. As discussed in [9], emission from such arrays has a strong angular dependence. Below we show measurements for the meander array at α = 45°, which corresponds to the emission maximum.

**Figure 7:**
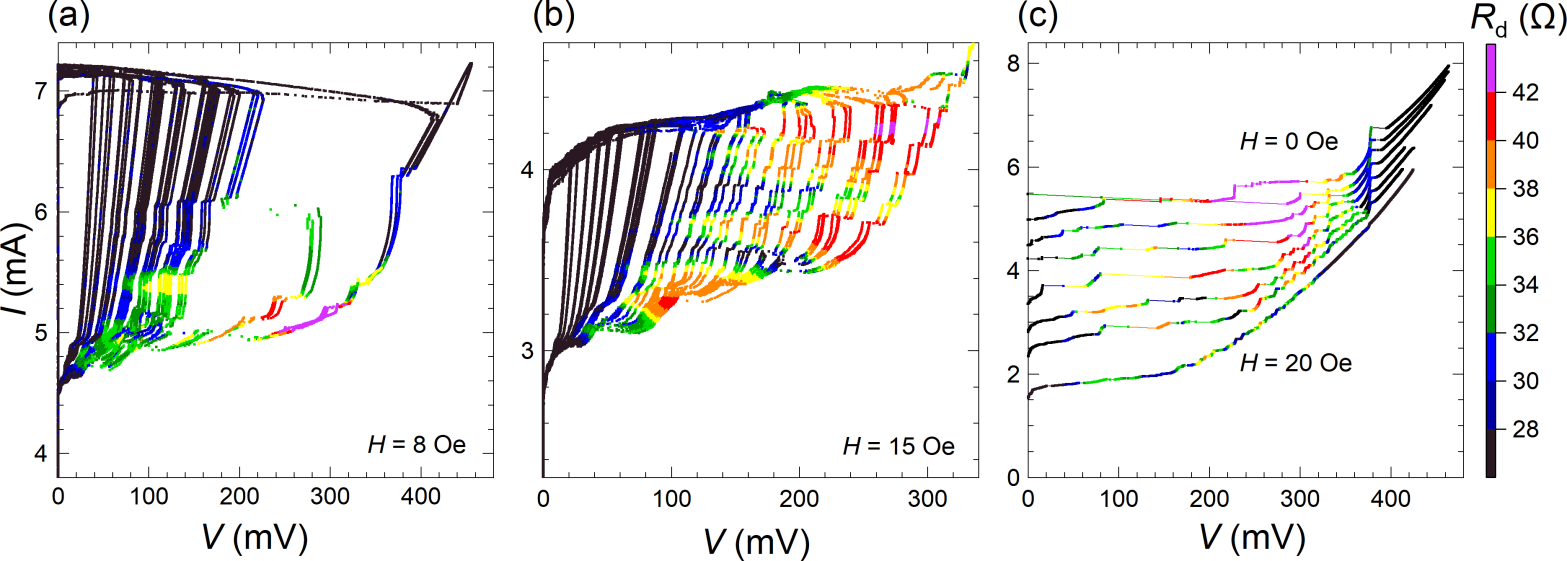
(a, b) The *I*–*V* characteristics (integrated oscillograms) of the meander array acquired at (a) *H* = 8 Oe and (b) 15 Oe at an angle of 45° with respect to the normal of the JJs. Panel (c) shows the return branch of the *I*–*V* characteristics (single swipe) at *H* = 0, 7, 9, 11, 13, 15, 17, and 20 Oe (from top to bottom). All *I*–*V* curves are color-coded by the simultaneously measured detector resistance *R*_d_ with the scale shown on the right side of panel (c). A correlated development of resonant step amplitude and the emitted power can be seen. Measurements are done at *T* = 2.4 K at the angle α = 45° between array and detector.

Figure 7 represents simultaneous measurements of the *I*–*V* characteristics and the detector response (represented by the color scale) for the meander array. Figure 7a and Figure 7b show two integrated oscillograms acquired in external fields of 8 and 15 Oe, respectively. Figure 7c shows the *I*–*V*s (single sweeps at the returning branch) for fields from 0 to 20 Oe at an angle of 45° with respect to the normal of the JJs. It is seen that the magnetic field strongly affects the emission. For example, at *H* = 8 Oe in Figure 7a, the main resonance is dominating, but, at *H* = 15 Oe in Figure 7b, many more smaller steps appear. The main resonance is not shown in Figure 7b in order to show details of the smaller steps, but it is still there, as can be seen from Figure 7c. The *I*–*V*s from Figure 7 reveal several emitting resonances, for which the emission power grows in a correlated manner with the step amplitude upon increasing the number of active JJs.

The linear array exhibits a qualitatively similar behavior, as reported in [9,34] and as shown in Figure 4. However, the presence of many nearby steps complicates unambiguous identification and analysis of specific cavity modes.

## Discussion

**Figure 8:**
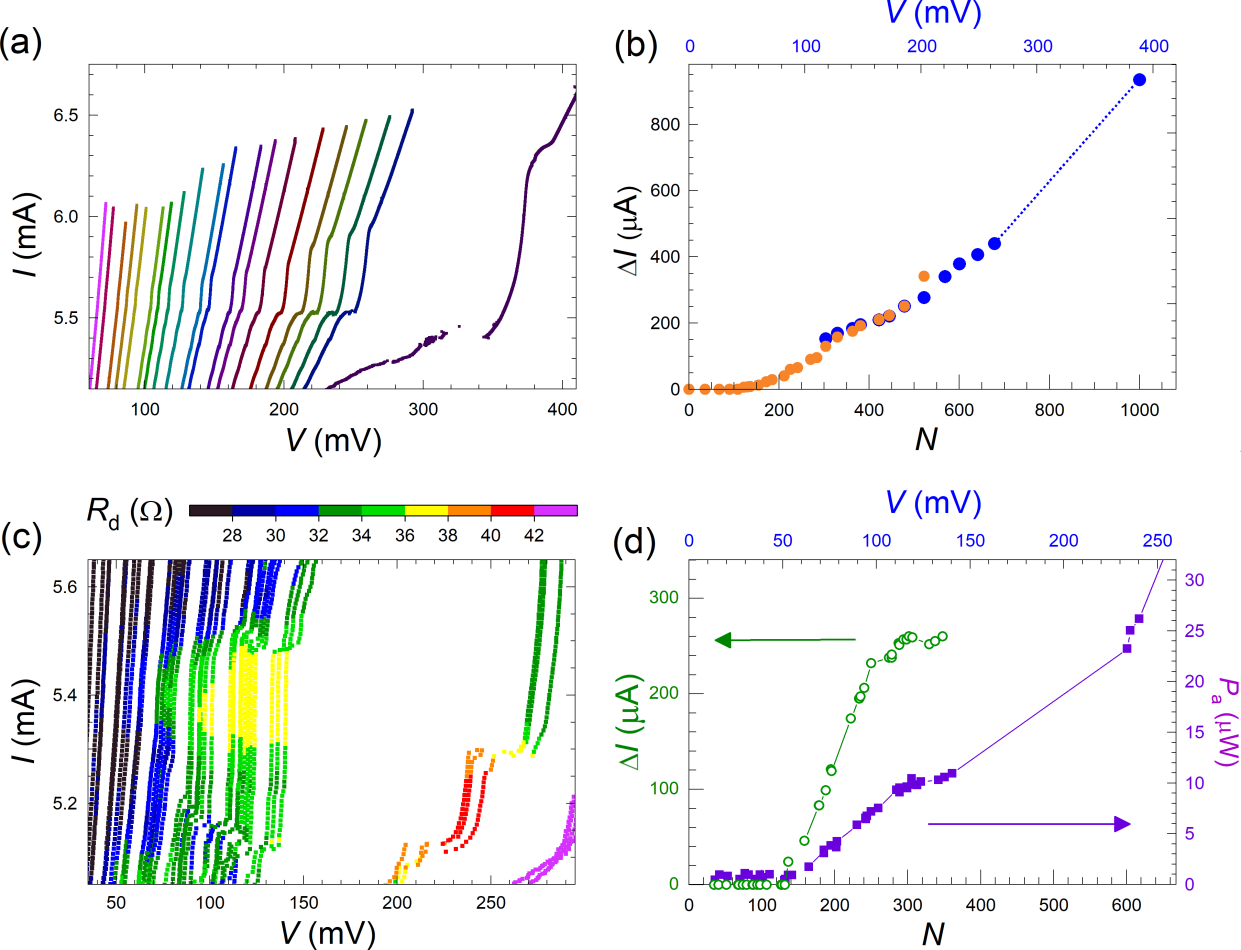
(a) Parts of the *I*–*V* characteristics of the meander array near the main resonance. (b) The main step amplitude, Δ*I*, as a function of the number of active junctions, *N*. Blue symbols are measured directly from the *I*–*V* characteristics, and orange symbols are obtained by integration of the resonant peak area in d*I*/d*V*. (c) Simultaneous transport and detection measurements of a secondary resonance from Figure 7a. (d) Step amplitude, Δ*I*, (olive open circles) and the detected (absorbed) power, *P*_a_, (purple solid squares) as a function of *N* for the secondary step from (c).

Figure 8 summarizes our main results, that is, the observation of collective excitation of surface plasmon resonances by large Josephson junction arrays and the correlated enhancement of EMW emission. Figure 8a shows a close-up on the *I*–*V* characteristics from Figure 3b for the meander array at the main resonance. Figure 8b shows the step amplitude as a function of the number of active JJs for this resonance. Blue symbols represent Δ*I* measured directly from the *I*–*V* characteristics. Orange symbols are obtained by integration of the areas of the peak in differential conductance, 
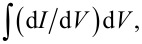
 taken from Figure 5b. The latter data set was multiplied with a calibration factor of 2.63 in order to merge it with direct measurements of Δ*I* in the range 300 *< N <* 500 where both methods are working well. A quasi-linear growth in Δ*I*(*N*) starting from the threshold number *N*_th_ ≃ 150 is observed. However, as can be seen from Figure 7a and Figure 7c, there is practically no emission at this most prominent step with *V*(*N* = 1000) = 389 mV, which corresponds to *f*_J_ ≃ 188 GHz, even when all *N* = 1000 JJs are active. This is not surprising because a non-emitting cavity mode is expected to have the highest quality factor due to the lack of radiative losses [19]. Therefore, this step is large because it is non-emitting.

Figure 8c shows the data for a secondary resonance from Figure 7a with a lower voltage and *f*_J_ ≃ 182 GHz. In Figure 8d, we plot the step amplitude, Δ*I* (olive, left axis), and the detected (absorbed) MW power, *P*_a_ (violet, right axis), as a function of *N* for the secondary mode from Figure 8c. The general behavior is similar, that is, a quasi-linear growth from a threshold number *N*_th_ ≃ 160. Up to *N* = 350, there is a direct correlation, *P*_a_ ∝ Δ*I*. However, at larger *N >* 600 we observe a superlinear upturn in the emission power, consistent with [9]. This is a likely indication of the global synchronization of the array, which leads to the coherent superradiant emission, for which *P*_MW_ should be proportional to Δ*I*^2^. The emitted power at *N >* 700 becomes so large that it saturates our detector. Therefore, unfortunately we can not confidently analyze further development of the mode.

**Figure 9:**
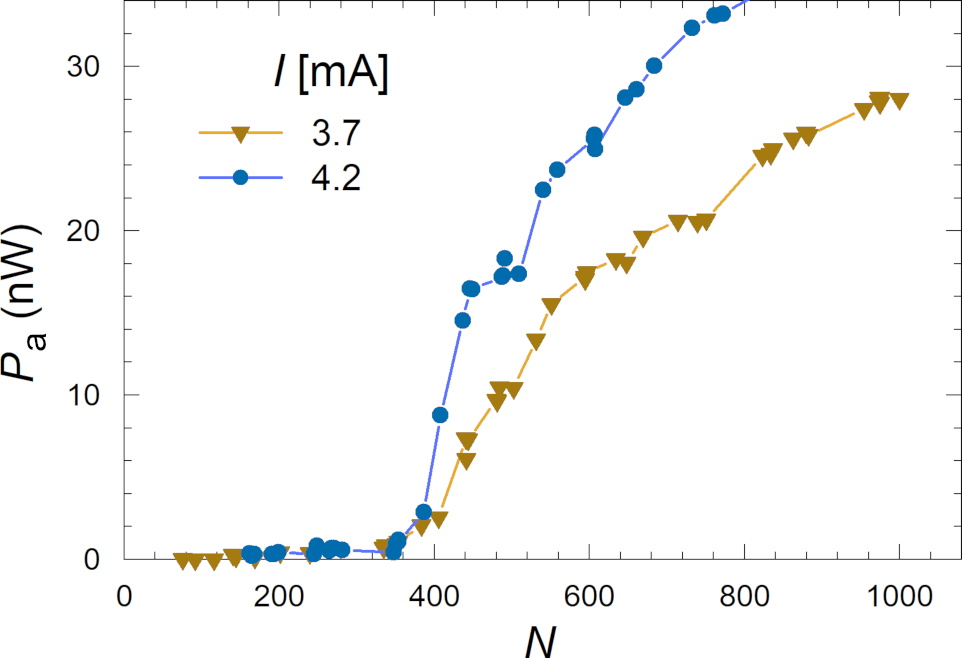
Detected power as a function of the number of active junctions for two modes from Figure 7b at bias currents of *I* = 3.7 mA (down triangles) and *I* = 4.2 mA (circles).

Generally, we observe that all cavity modes are behaving similarly, that is, Δ*I* and *P*_a_ increase in a quasi-linear manner with *N*, above some threshold number *N*_th_. This clearly shows a collective nature of the excited resonance. Yet, there are some differences. For example, some modes emit strongly and some do not emit at all. Such behavior is characteristic for constructively and destructively interfering coherent states [19]. Also, the threshold number is individual for each mode. The large variety of modes and their differences can be seen in Figure 7b. In Figure 9, we analyze the detected power vs *N* for the two most distinct emitting modes from Figure 7b at different biases *I* = 3.7 mA (down triangles) *I* = and 4.2 mA (circles). Here, the threshold number is significantly larger, *N*_th_ ≃ 380, and the power does not grow linearly but increases abruptly for *N > N*_th_.

The results presented here are consistent with several previous works. A linear increase of a resonant step amplitude with increasing junction number has been observed in [30–31]. A threshold behavior for excitation of a common resonance has been reported in [8,15]. However, the ability to individually switch JJs in very large arrays allows us to observe those effects with an unprecedented clarity. This facilitates unambiguous interpretation of synchronization and emission mechanisms from such arrays, in which JJs are indirectly coupled via a common resonator. Combining our results with earlier observations, we can make the following conclusions on the mechanisms of formation of collective resonant steps, emission, and synchronization in large JJ arrays:

(I) The mechanism for the formation of resonant steps in the *I*–*V* is similar to that behind Shapiro steps with the only difference that the corresponding MW current, *I*_MW_, originates from the cavity mode in the array rather than from an external source. Therefore, the amplitude of the step, Δ*I*, is proportional to the critical current and modulates together with it as a function of magnetic field, as demonstrated in Figure 2. At small step amplitudes, Δ*I* ≪ *I*_c0_, the cavity mode current is directly proportional to the step amplitude, *I*_MW_ ≃ *a*Δ*I* with *a* ≈ 1 (see section 11.3 in [38]).

(II) The absence of emission at *N < N*_th_ shows that the emission in this case is not caused by direct interference of electromagnetic fields from individual JJs. The JJs in our study have an overlap (parallel-plate capacitor) geometry. They are characterized by a large impedance mismatch and a very low emission efficiency [19]. The emission is facilitated by the long (approx. 1 cm) electrode, which acts as a matching travelling wave antenna [9]. The role of the JJs is just to pump oscillations, *I*_MW_, in the antenna. Therefore, the quadratic increase of the emission power as a function of the number of active JJs [8,30,42] in this case is not due to direct interference of radiation fields from individual JJs (which are negligible due impedance mismatch) but is the consequence of the linear *I*_MW_(*N*) ∝ Δ*I*(*N*) ∝ *N* dependence. The emission power from the antenna in this case remains quadratic: *P*(*N*) = *I*_MW_^2^(*N*)/*Z* ∝ *N*^2^, where *Z* is the corresponding microwave impedance. However, generally *I*_MW_(*N*) can be nonlinear, in which case *P*(*N*) is non-quadratic. Indeed, the nonlinear *I*_MW_(*N*) dependence often appears in corresponding numerical simulations [33]. Furthermore, the simple analysis above assumes a perfect phase-locking of the array to a single cavity mode, which is not always the case. During the experiments we observed switching between nearby modes, which prevents us from a straightforward analysis of the linear array. Presumably, such instability and incomplete phase-locking leads to a linear dependence *P*_a_(*N*) ∝ Δ*I*(*N*), which is shown in Figure 8d up to *N* ≃ 430. An improvement of phase-locking at larger *N* leads to the boost in the emission power, as also reported in [9]. Yet, for JJs coupled via a cavity mode, even a perfect parabolic dependence *P* ∝ *N*^2^ is not the consequence of direct superradiant emission from *N* JJs. It should rather be referred to as a collective cavity mode emission (emission by a cavity mode pumped collectively by a phase-locked array of oscillators). This is still a coherent phenomenon, but the emission occurs from a single source, namely the antenna formed by the electrode.

(III) The synchronization: the overlap-type JJs in the studied arrays are separated by 12 μm, which is much larger than the London penetration depth. This precludes direct interaction between the JJs [28]. Therefore, JJs are coupled only indirectly via common cavity modes, corresponding to the formation of standing surface plasmon waves along the electrode [34]. The standing wave imprints its order on Josephson junctions in the array. This requires a critical amplitude, which translates into a threshold number of active JJs for excitation of the cavity resonance [32–33].

## Conclusion

We have studied large arrays containing up to 1000 Nb/Nb*_x_*Si_1−_*_x_*/Nb Josephson junctions. By applying a small magnetic field, we managed to acquire a large variety of dynamic states with different numbers of active junctions in the oscillating resistive state. This allowed for a detailed analysis of the collective phenomena that take place in the arrays. We reported a gradual development of cavity mode resonances in the arrays upon sequential switching of JJs into the oscillating state. We show that a threshold number of JJs, *N*_th_ ≈ 100, is required for excitation of such resonances. Above the threshold, the amplitude of resonant steps in the *I*–*V* characteristics grows in a quasi-linear manner with the number of active JJs. We employ an external microwave detector to measure the electromagnetic wave emission from the arrays. It is observed that the emission power is correlated with the amplitude of the resonant steps in the *I*–*V* characteristics.

Our observations clearly reveal the collective, indirect mechanism of interjunction coupling. Studied junctions are of the overlap type and are separated by a distance of 12 μm, that is, one hundred times larger than the London penetration depth in Nb. This precludes direct interactions between them. Nevertheless, they can be effectively synchronized via the indirect coupling mechanism mediated by the extended centimeter-long electrodes in the arrays, which act as transmission lines for surface plasmon-type electromagnetic waves. Therefore, they both support collective cavity resonances and act as matching antennas for microwave emission.

Our observations imply that cavity modes in the electrodes are pumped collectively by the junctions, which are in turn mutually phase-locked by the modes. This provides a positive feedback mechanism, which allows for the synchronization of large arrays without direct interjunction interaction. The electromagnetic wave emission in this case is facilitated by the cavity modes in the large resonator outside the junctions formed by the electrodes, rather than by direct emission from the junctions. We conclude that such indirect coupling is effective for the synchronization of very large arrays.
